# Endoscopic stent placement with laparoscopic stent fixation in a patient with obstruction at a gastrojejunostomy anastomosis site

**DOI:** 10.1186/s40792-023-01699-4

**Published:** 2023-06-29

**Authors:** Kiwa Son, Koji Shindo, Kenoki Ouchida, Taiki Moriyama, Koji Tamura, Kinuko Nagayoshi, Yusuke Mizuuchi, Naoki Ikenaga, Kohei Nakata, Masafumi Nakamura

**Affiliations:** Department of Surgery and Oncology, Graduate School of Medical Sciences, Kyushu University, Fukuoka, Japan

**Keywords:** Laparoscopic stent fixation, Stent migration, Anastomosis site obstruction, Gastrojejunostomy, External alimentary tract compression, Recurrent malignancy, Palliative surgery

## Abstract

**Background:**

Palliative endoscopic stent placement may be considered in patients with malignant gastrointestinal obstruction. Stent migration is a potential complication, particularly for those placed at a surgical anastomosis or across a stricture caused by extra-alimentary tract factors. We report a patient with left renal pelvis cancer and gastrojejunostomy obstruction who underwent endoscopic stent placement and laparoscopic stent fixation.

**Case presentation:**

A 60-year-old male with peritoneal dissemination of a left renal pelvis cancer was admitted for treatment of upper gastrointestinal obstruction. A laparoscopic gastrojejunostomy had been previously performed for cancer invasion of the duodenum. Imaging showed gastroduodenal dilation and impaired passage of contrast medium through the efferent loop of the gastrojejunostomy. Gastrojejunostomy anastomosis site obstruction due to dissemination of left renal pelvis cancer was diagnosed. Conservative treatment failed and endoscopic stent placement with laparoscopic stent fixation was performed. After surgery, the patient was able to tolerate oral intake and he was discharged without complications. The patient gained weight and was able to resume chemotherapy, indicating the procedure was effective.

**Conclusions:**

Endoscopic stent placement with laparoscopic stent fixation for malignant upper gastrointestinal obstruction appears effective in patients with a high risk of stent migration.

## Background

In patients with gastrointestinal obstruction due to an unresectable malignant tumor, stent placement is frequently performed when surgery is not indicated [1]. Stent migration is one of the most frequent stent-related complications; others include intestinal obstruction, bleeding, and perforation [2, 3]. Endoscopic suture fixation and stent fixation using an over-the-scope clip can prevent stent migration in patients with esophageal cancer [4–6]. However, to the best of our knowledge, laparoscopic stent fixation (LSF) has not been previously reported. We report a patient with disseminated cancer of the left renal pelvis and obstruction at the site of a gastrojejunostomy (GJ) anastomosis in whom we performed endoscopic stent placement with LSF.

## Case presentation

**Fig. 1:**
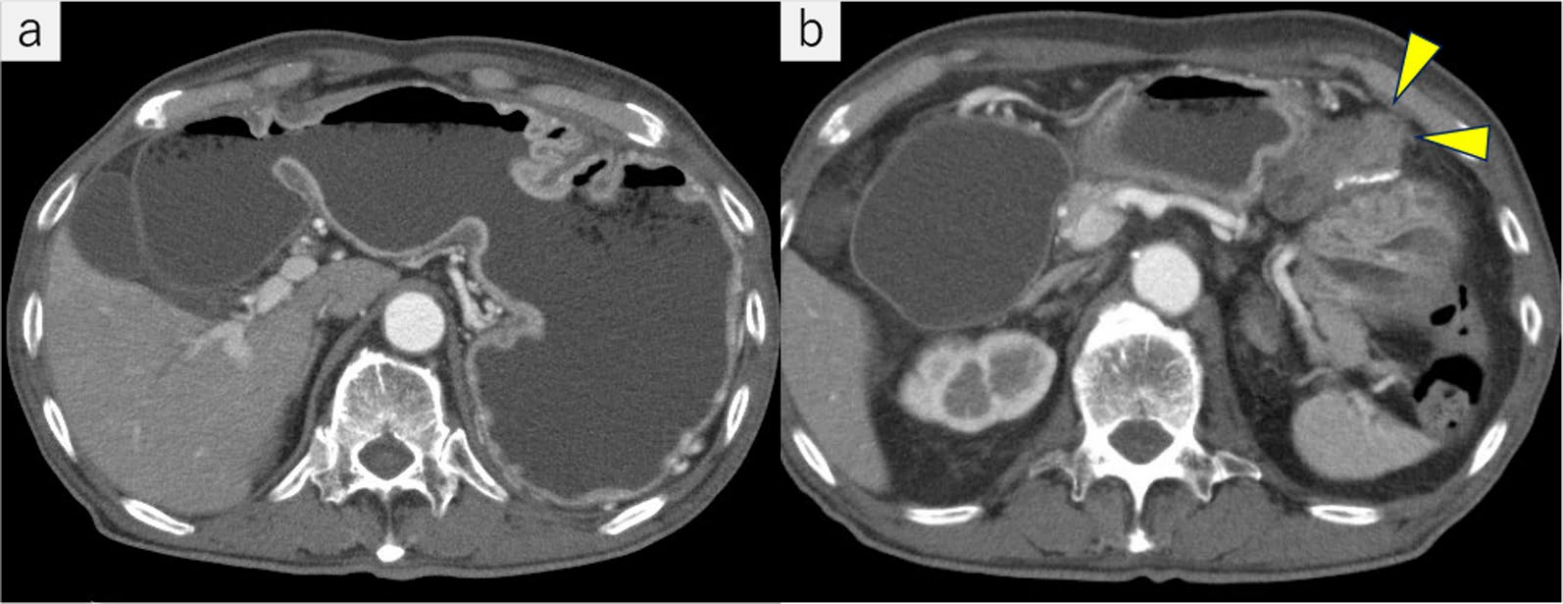
Computed tomography on admission. **a** Stomach and duodenum are prominently dilated. **b** Obstruction at the gastrojejunostomy anastomosis was caused by disseminated left renal pelvis cancer (arrowhead)

**Fig. 2:**
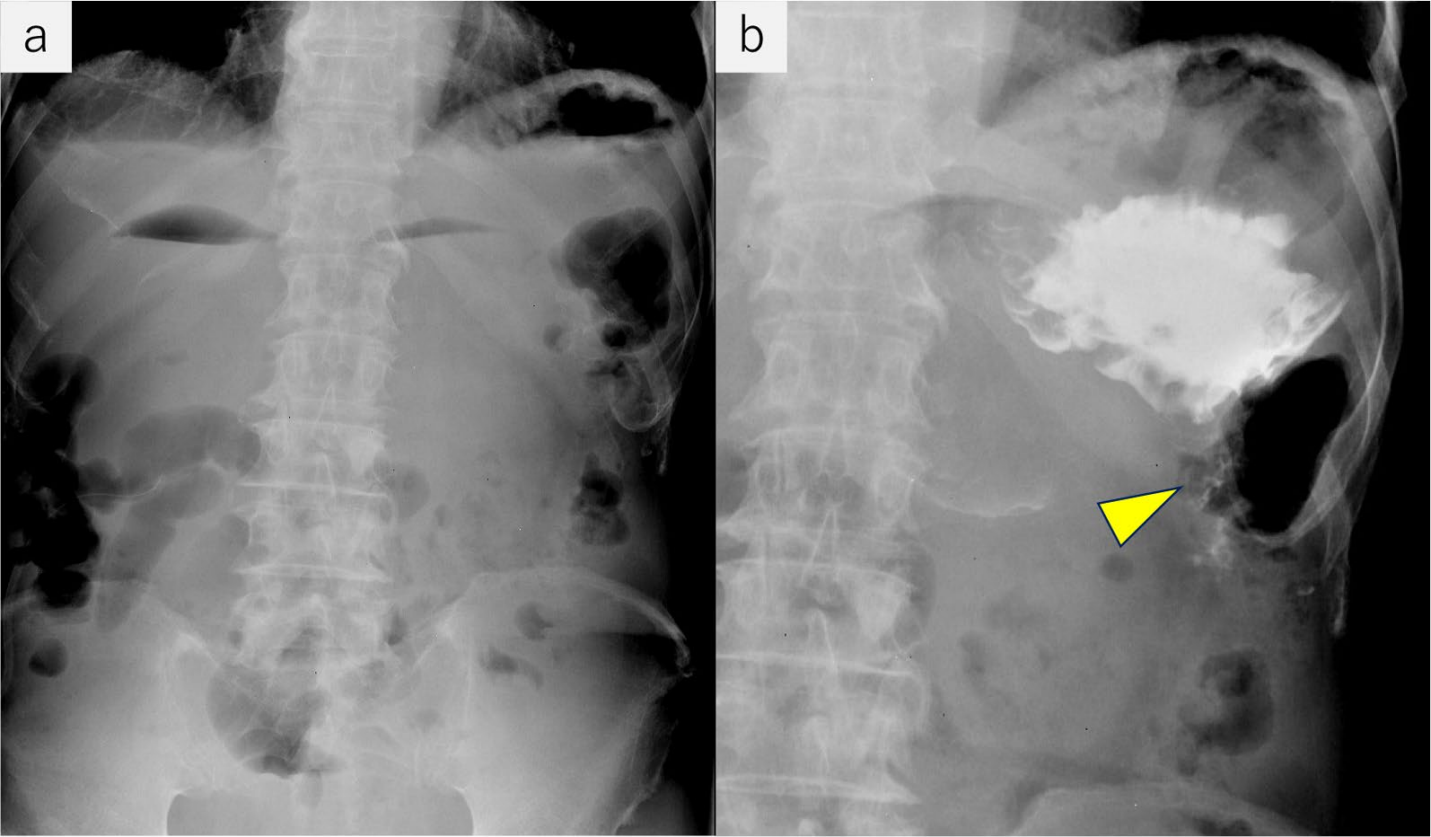
Upper gastrointestinal fluoroscopy before surgery. **a** Niveau formations were visualized in the stomach, duodenum, and proximal jejunum. **b** Contrast medium was retained in the stomach; only a small volume passed into the efferent loop of the gastrojejunostomy (arrowhead)

**Fig. 3:**
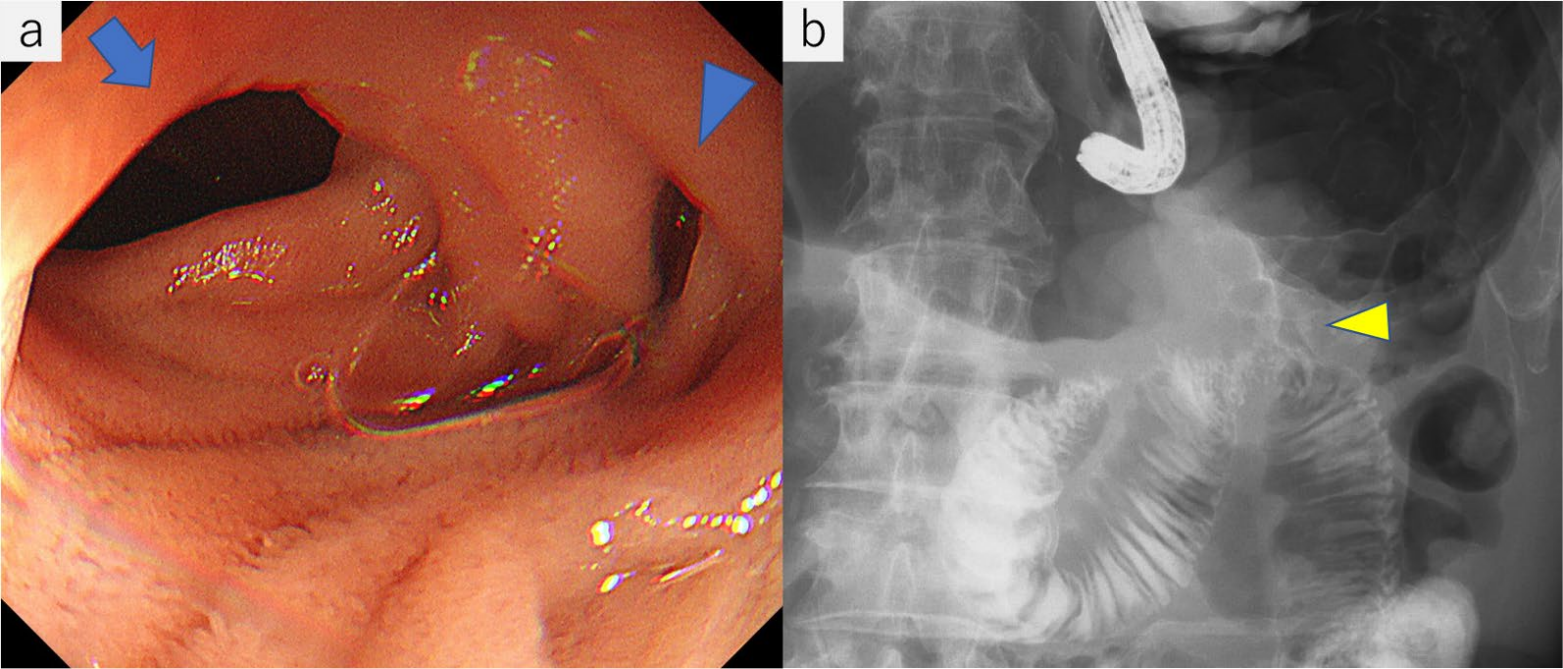
**a** Endoscope was able to pass with only slight resistance. The afferent loop (arrow) and efferent loop (blue arrowhead) had no mucosal abnormalities. **b** Stricture of the gastrojejunostomy was revealed on fluoroscopy (yellow arrowhead)

A 60-year-old male with cT4N0M1 stage IV left renal pelvis cancer with peritoneal dissemination and a history of laparoscopic GJ performed for cancer invasion of the duodenum presented with vomiting and was admitted to the hospital. His renal cancer had been treated with chemotherapy as radical surgery was not indicated. Computed tomography of the abdomen showed significant gastroduodenal dilation. GJ obstruction (Fig. 1a, b) and renal cancer progression were suspected. On fluoroscopic examination, the stomach was dilated and contrast medium did not pass into the efferent loop of the GJ (Fig. 2a, b). Upper gastrointestinal endoscopy revealed no abnormalities of the mucosal surface. The scope was able to pass through the efferent loop and no obvious hard stricture was found. Because fluoroscopy showed a stricture of the GJ anastomosis but the endoscopic findings were unremarkable, we suspected the stricture was caused by external compression of the alimentary tract (Fig. 3a, b). GJ anastomosis site obstruction owing to disseminated left renal pelvis cancer was diagnosed.

**Fig. 4:**
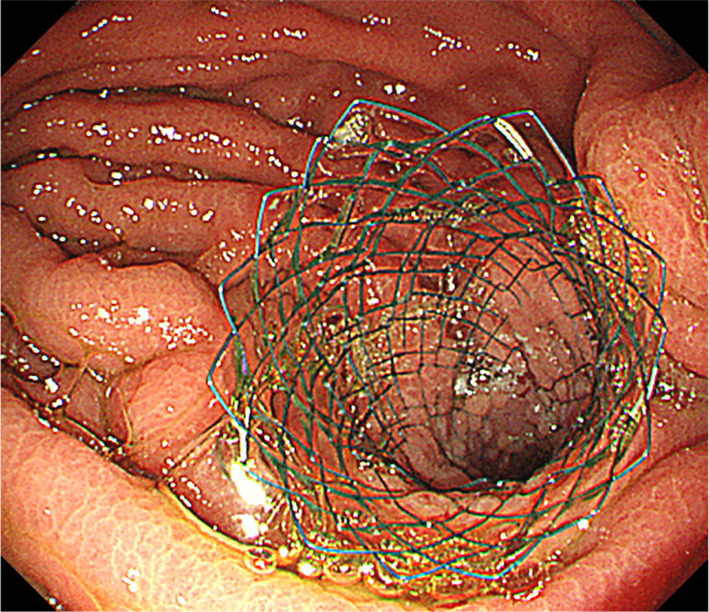
Self-expanding metal stent was placed into the stomach across the anastomosis to the efferent loop

**Fig. 5:**
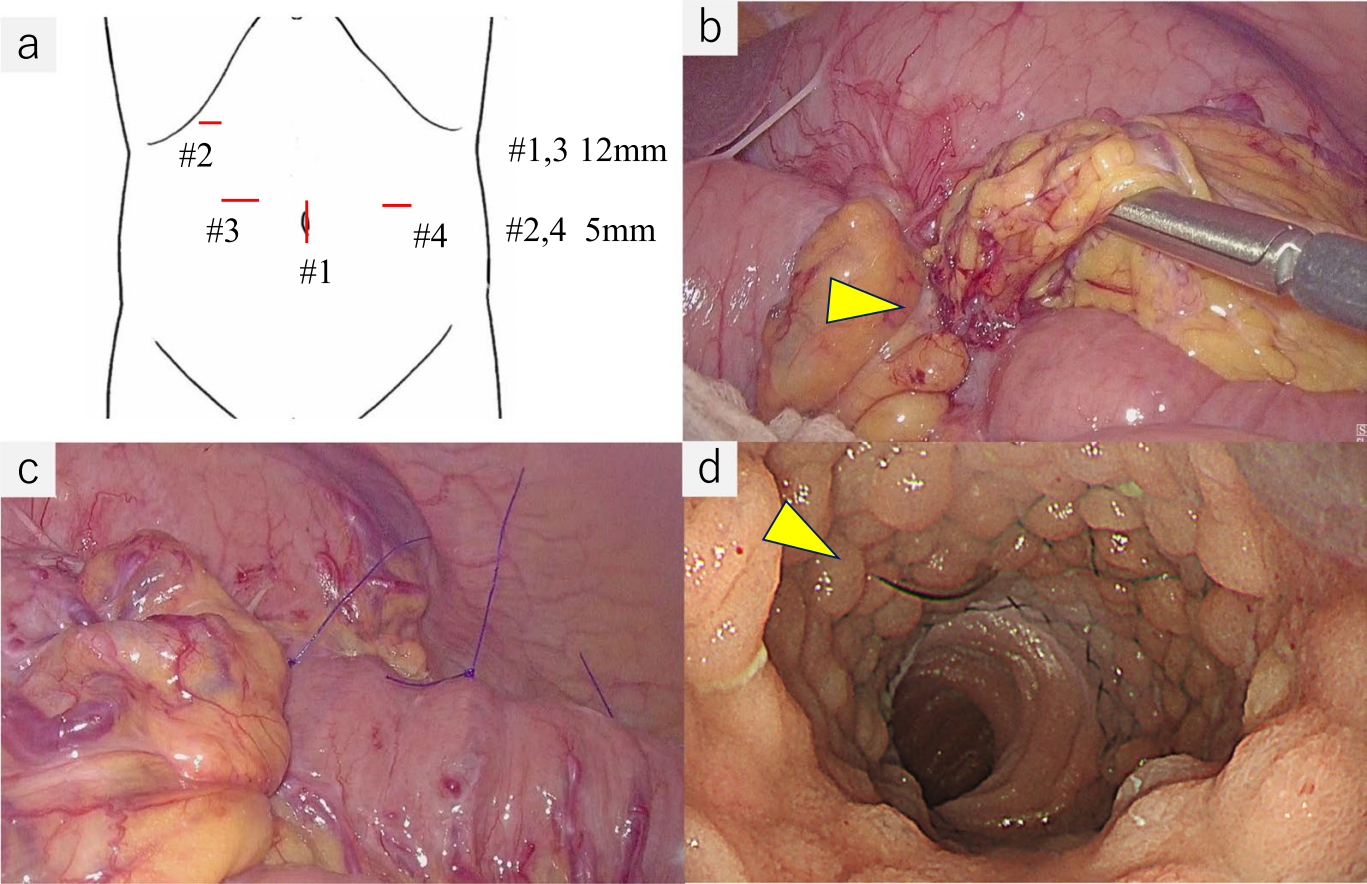
**a** Port placement. **b** Hard nodule of the greater omentum was adherent to the gastrojejunostomy anastomosis (arrowhead). **c** Stent was fixed using three 3–0 prolene sutures placed at 3 cm intervals. **d** Fixation was confirmed using intraoperative endoscopy (arrowhead)

Conservative treatment including tube decompression failed and endoscopic stenting was considered; however, we were concerned about stent migration, because the stricture was not hard. Therefore, endoscopic stent placement was performed in conjunction with LSF. First, a self-expanding metal stent was placed endoscopically into the stomach across the stricture under general anesthesia (Fig. 4). Then, LSF was performed using four laparoscopy ports (Fig. 5a). Multiple peritoneal nodules were observed in the upper abdominal cavity. A hard nodule of the greater omentum was adherent to the GJ anastomosis site, which was thought to be the cause of the stricture (Fig. 5b). The efferent loop with the stent was fixed using three 3–0 prolene sutures placed at 3 cm intervals (Fig. 5c). Fixation was confirmed using intraoperative endoscopy (Fig. 5d). Gastrostomy was performed at the end of the operation for gastric decompression and enteral nutrition administration as a precautionary measure. Operation time was 125 min. Intraoperative bleeding was minimal.

**Fig. 6:**
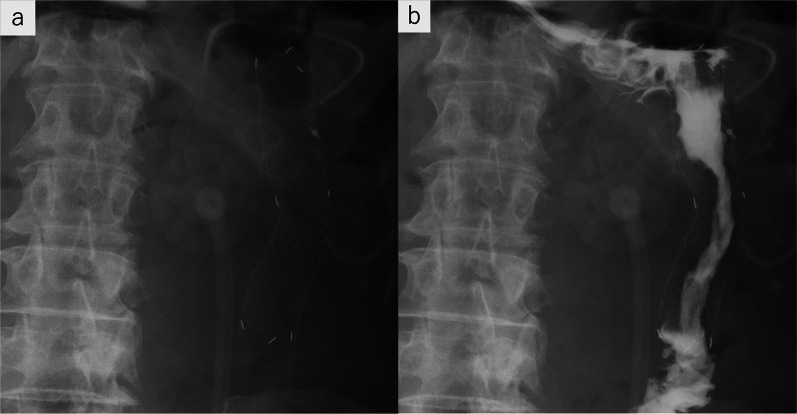
Upper gastrointestinal fluoroscopy after surgery. **a** Stent was open and did not migrate. A clip beside the stent was placed endoscopically during surgery to mark the best site to open the stent. **b** Contrast medium passed through the efferent loop of the gastrojejunostomy. No stricture was observed

**Fig. 7:**
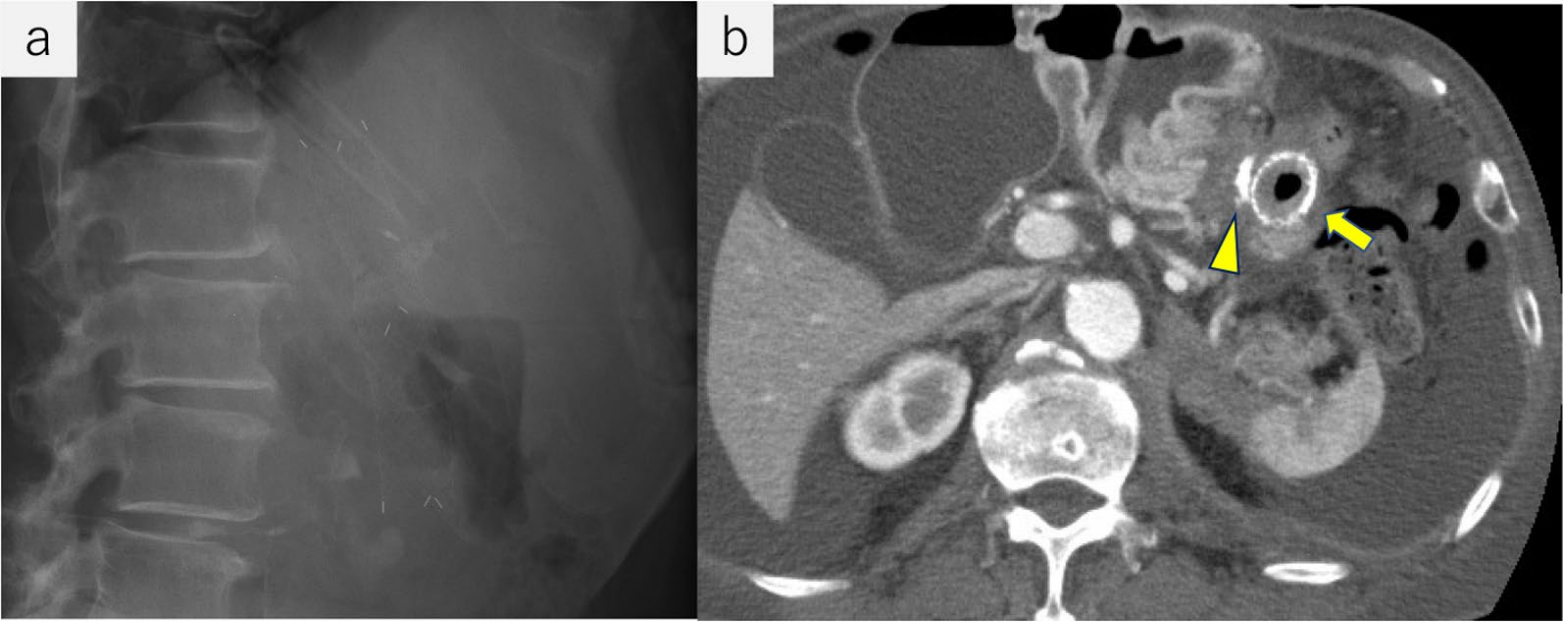
**a** Plain radiography 47 days after surgery confirmed the stent was open and had not migrated. **b** Computed tomography 88 days after surgery showed the stent was open and located at the gastrojejunostomy anastomosis (arrow). A staple at the site is visible (arrowhead). However, ascites had increased and the cancer had progressed

After surgery, fluoroscopic examination showed release of the stricture and appropriate stent placement (Fig. 6). Oral intake was initiated on postoperative day (POD) 3. The patient was discharged on POD 17 without complications. He continued chemotherapy after discharge and his body weight had increased by 3 kg on POD 30, indicating procedural success. Plain radiography on POD 47 (Fig. 7b) and computed tomography POD 88 (Fig. 7b) showed the stent was open and had not migrated. The patient died of renal cancer 3 months after stent placement/LSF without symptoms of upper gastrointestinal obstruction.

## Discussion

In the palliative setting, surgical GJ may be considered to treat gastric outlet obstruction in patients with > 2 months life expectancy and good functional status [7]. Laparoscopic GJ is favored over open, because it is less invasive and associated with a shorter hospital stay [8]. Since the introduction of endoscopic ultrasound-guided gastroenterostomy [9], several studies have shown its effectiveness [10–13]; however, it is not yet widely performed. Other reports have described interventions for obstruction caused by GJ anastomosis stenosis [14–18]. To the best of our knowledge, intervention for obstruction caused by factors outside the alimentary tract following GJ, as in our patient, has not been reported.

Gastrointestinal obstruction by a recurrent malignancy after palliative GJ usually reflects tumor progression and poor general condition; rarely is it an indication for further surgery [19]. Repeated surgeries for recurrent malignancy could cause significant morbidity and mortality. In our patient, endoscopic stenting was selected, because he had a history of palliative GJ. However, the success rate of endoscopic stenting for anastomotic stricture is lower than that for gastric outlet or duodenal obstruction [20]. In addition, his stricture was caused by extra-alimentary tract compression, not locally recurrent gastric or intestinal cancer, and the scope was able to pass through. In general, stents do not migrate when the stricture was severe, but stent migration may have been more likely in such a case of soft stricture.

The role of colonic stents in treating obstruction in patients with extracolonic malignancy is controversial [21]. Success rates vary between reports [22–24]. In a study of patients with malignant colon obstruction undergoing colonic stenting, stent migration was more likely in patients with extracolonic malignancy than those with colon cancer; furthermore, the clinical success rate of stenting was lower in patients with extracolonic malignancy (20% vs. 89%) [22]. Our patient’s stricture was caused by external pressure from his left renal pelvis cancer. In addition, the mucosa at the GJ anastomosis appeared normal on endoscopy. Both suggested that the risk of stent migration was high. Chemotherapy after stent placement is also associated with stent migration, as chemotherapy reduces the tumor burden [20]. Our patient was scheduled for chemotherapy after stenting, which was another risk factor for stent migration.

Endoscopic stent suturing and over-the-scope clipping are promising measures to prevent stent migration which have a high success rate [4–6]. However, these techniques are not yet widely used. The risk of stent migration was high in our patient for the reasons mentioned above, plus his stricture was soft enough to allow passage of an endoscope. Therefore, we performed LSF, a technique which has not been previously reported. After surgery, he was able to tolerate oral intake, gain weight, and continue chemotherapy.

LSF can be a suitable procedure for patients with the risks of stent migration as previously mentioned. It can offer an alternative option for palliative treatment of upper gastrointestinal obstruction, especially when endoscopic fixation is not feasible.

## Conclusions

Endoscopic stent placement with LSF for malignant upper gastrointestinal obstruction appears effective in patients with a high risk of stent migration. Future studies are warranted to further investigate and determine its durability.

## Data Availability

Not applicable.

## Abbreviations

LSF: Laparoscopic stent fixation
GJ: Gastrojejunostomy
POD: Post-operative day
